# 

**Published:** 1873-03

**Authors:** 


### TABLE OF CONTENTS.

### ORIGINAL COMMUNICATIONS.

The Corset in its Relations to Uterine Diseases. By V. H. Taliaferro, M.D., Professor of Di eases of Women in Atlanta Medical College..... 683
Notes of Two Cases of Tetanus. By B. S. Herndon, M.D................ 694
Atlanta Academy of Medicine. Jas. B. Baird, M.D., Reporter........... 697
Surgical Clinic of Prof. Westmoreland. Reported by C. M. Hill, M.D. 704
Abstracts from German Journals. By Ch. Rauschenberg, M.D......... 708

### SELECTIONS.

Keloids. By Wm. Augustus Brown, M.D................................ 719
On Death from Chloroform. (Extract from Lecture by Dr. Erichsen.)... 726
Death of the Emperor Napoleon.......................................... 728
Paralysis of Various Organs, Due to Disease of the Heart.............. 730

### EDITORIAL AND MISCELLANEOUS.

Close of the Volume.................................................. 731
Georgia Medical Association.......................................... 732
On the Treatment of Tremor........................................... 733
Subcutaneous Injections of Ergotine in Uterine Fibroids................ 734
Georgia Medical Association.......................................735, 740
American Medical Association......................................... 737
To the Medical Profession............................................ 740

